# Biologic anti-IL17 drugs in erythrodermic psoriasis

**DOI:** 10.1016/j.jdin.2024.05.007

**Published:** 2024-06-22

**Authors:** Alessandro Falco, Cristina Mugheddu, Jasmine Anedda, Laura Pizzatti, Alice Tatti, Brunella Conti, Laura Atzori

**Affiliations:** Dermatology Clinic, Department Medical Sciences and Public Health, University of Cagliari, Cagliari, Italy

**Keywords:** anti-IL17, biologics, Erythrodermic psoriasis, ixekizumab, psoriasis, secukinumab, therapy

## Abstract

**Background:**

Erythrodermic psoriasis (EP) is a potentially life-threatening disease, and there is currently no consensus regarding its optimal treatment. Biological drugs approved for Psoriasis Vulgaris treatment have been used as alternatives to traditional medications.

**Objective:**

To evaluate the clinical response and tolerability of anti- interleukin 17 (IL17) biologic drugs during a 2-year-follow-up.

**Methods:**

This was a retrospective prospective study. EP cases, defined as >75% body surface area involvement, in patients ≥18 years old treated with anti-IL17 for at least 6 consecutive months were enrolled and then followed until 104 weeks. Patient characteristics, overall clinical responses, Psoriasis Area Severity Index score changes, and adverse events were analyzed.

**Results:**

Sixteen patients met the criteria, of which 50% had achieved the Psoriasis Area Severity Index 100 response at week 12 and in 93.7% at week 24. In the prospective observation of the cohort, 87.5% were still in remission at week 52 and 81.25% at 104 weeks, without adverse events. The 3 patients in whom the treatment was interrupted lost efficacy and were switched to other therapies.

**Limitations:**

Only descriptive analysis was conducted due to the limited number of patients.

**Conclusions:**

A satisfactory long-term clinical response without adverse effects was observed in this case series, suggesting the interest of anti-IL17 in EP treatment.

**Capsule Summary**

• There is no consensus regarding the best treatment algorithm for Erythrodermic psoriasis. In this case series, treatment with anti-IL 17 drugs demonstrated positive response without adverse events.
• Alternatives to conventional systems are warranted as they often present contraindications or side effects, and anti-IL 17 drugs candidate are a promising option.

## Introduction

**Fig 1:**
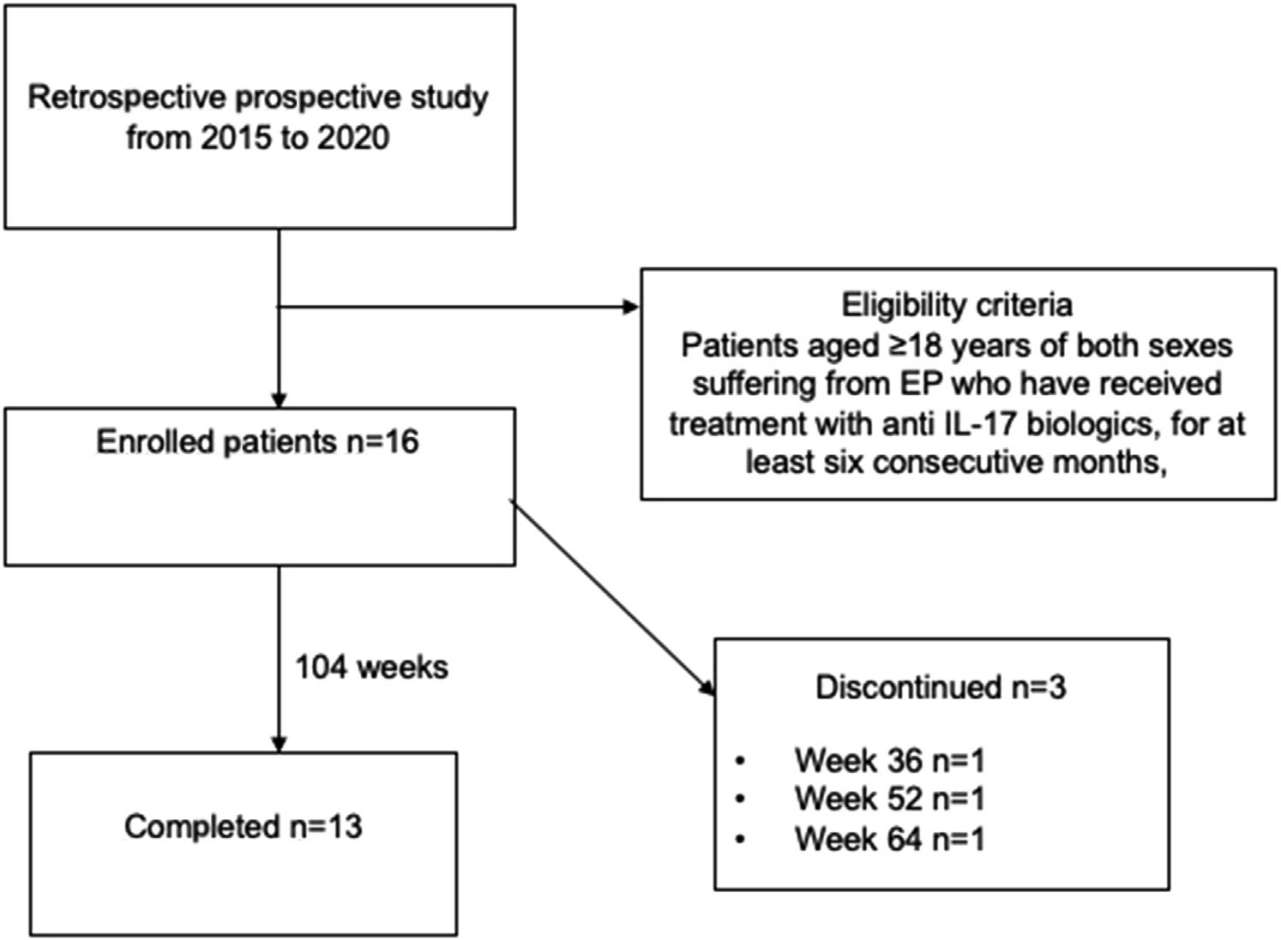
Flow diagram of the study design.

Erythrodermic psoriasis (EP) is a rare, difficult-to-treat variant of psoriasis associated with a potentially life-threatening severe clinical course.^1^ Characterized by widespread erythema affecting almost the entire body surface (body surface area [BSA] from 75% to more than 90%), EP is among the most common causes of erythroderma, responsible for approximately 25% of all cases. This condition generally develops rapidly or more gradually (from days to weeks) in patients already suffering from poorly controlled psoriasis vulgaris and resolves with desquamation and exfoliation (Fig 1). The prevalence is estimated to be 1% to 2.25%, with a 3:1 male to female ratio.^2^

Several factors are considered as EP triggers: sudden withdrawal of psoriasis systemic drugs, such as corticosteroids and methotrexate; intake of medications, including lithium and antimalarial drugs; and systemic infections.^2^

Skin itching or pain is accompanied by systemic symptoms, including fever, chills, malaise, tachycardia, and arthralgia. Leukocytosis, eosinophilia, and anemia are often found on laboratory tests. Thus, EP patients may experience severe complications, including electrolyte imbalances, hypoalbuminemia, and higher susceptibility to skin infections.^3^

Owing to the rarity of this condition, there is currently no consensus regarding the best treatment algorithm for EP.^4^ In 2011, the National Psoriasis Foundation provided consensus guidelines for the first- and second-line treatment of EP, stating that cyclosporine and infliximab are recommended for unstable patients, while methotrexate and acitretin can be used for clinically stable patients.^5^

However, conventional systemic drugs have contraindications or side effects. Increasing experience with biological drugs suggests that the indications for EP treatment should be extended.

Preliminary reports support the use of 2 anti-IL17 A agents, secukinumab and ixekizumab in EP patients, emphasizing the rapidity of action and achievement of a satisfactory long-term clinical response.^6^, ^7^, ^8^ Interleukin 17A is a pro-inflammatory cytokine secreted by Th17 cells, natural killer cells, mast cells, and neutrophils, and plays a crucial role in severe widespread psoriasis.^9^

This study aimed to evaluate the results of anti-IL17 biologic drugs (secukinumab and ixekizumab) administration in EP patients treated at the Psoriasis Center of the Dermatology Clinic of the University of Cagliari.

## Materials and methods

This retrospective prospective study was approved by the Ethical Committee of Azienda Ospedaliera Universitaria Cagliari in July 2020 (PER-PUGIL 17- Prot. No. PG/2017/5575).

EP was defined as >75% of BSA involvement with inflammatory erythema and scaling at baseline.

Patients aged ≥18 years of both sexes suffering from EP who have received treatment with anti IL-17 biologics, for at least 6 consecutive months, referred to the Dermatology Clinic of the University of Cagliari from 2015 to 2020 were included in this study.

The following exclusion criteria were used to exclude patients: EP patients under 18 years of age, treated with conventional or other biological drugs, and treated with anti-IL-17 drugs for less than 6 months.

All data from the medical records of the Psoriasis Center of the Dermatology Clinic of the University of Cagliari were recorded in a dedicated database for anonymization by assigning an alphanumeric code. Before receiving therapy and every 6 months during follow-up, all erythrodermic patients underwent laboratory evaluations that included complete blood count, liver and kidney function, hepatitis B and C markers, human immunodeficiency virus, and quantiferon. Additionally, malignancies were ruled out by mammography and PAP-test for women and Prostate Specific Antigen assays for men, which were repeated every 12 months.

Patient demographics and characteristics, including sex, age, smoking habits, comorbidities (hypertension, dyslipidemia, and diabetes), family history of psoriasis, age at onset, and presence of psoriatic arthritis, were collected.

Features of the erythrodermic form of psoriasis were also evaluated, including age at onset, whether it was an evolution of psoriasis vulgaris or de novo onset, and the presence of a possible trigger.

The choice of the anti-IL17 drug followed the current clinical practice in Italy. The Italian Agency for Drug Approval (AIFA) introduced secukinumab to the market approximately 1 year before ixekizumab became available. Thus, the majority of patients received subcutaneous (SC) injections of secukinumab on a standard regimen (300 mg at weeks 0, 1, 2, 3, and 4 in the induction dosing period, followed by 300 mg every 4 week as maintenance), and a smaller cohort received 160 mg of ixekizumab (2 80 mg SC injections at week 0, then 80 mg every 2 weeks for 12 weeks, and a maintenance dose of 80 mg SC every 4 weeks).

The major outcome of the study was the clinical response to the anti-IL17 drugs secukinumab and ixekizumab, as defined by Psoriasis Area Severity Index (PASI) assessment during EP treatment at week 12.

Secondary endpoints included sustained clinical responses at 24, 36, 52, and 104 weeks and tolerability profiles.

Only descriptive analyses using row numbers and percentages were conducted due to the limited number of patients included.

## Results

A total of 16 patients were enrolled: 12 males (75%) and 4 females (25%) (male to female ratio, 3:1). The mean age at onset was 52.5 years (range 26–84), 15 patients (93.7%) had a previous diagnosis of psoriasis vulgaris evolved into a generalized erythrodermic form. The mean age at onset of psoriasis vulgaris was 37.5 years (range 10–83), only 25% of patients had familiarity for the disease. Three patients (18.7%) were affected with psoriatic arthritis. Analyzing comorbidities, it was found that 5 patients (31.2%) had hypertension and 4 had dyslipidemia (25%). Additionally, 25% of the patients included in the study were smokers.

In 4 patients (25%), it was possible to identify a trigger: 2 patients developed the erythrodermic form following infectious episodes (in the first case it was pharyngotonsillitis; in the second pneumonia that required oral steroid therapy). In 2 other patients, erythroderma developed after the discontinuation of a systemic drug (in one case, the patient had to discontinue infliximab owing to elevated transaminase levels, while another patient discontinued methotrexate owing to poor compliance caused by work-related issues). Conversely, in 12 patients (75%), no trigger was found.

All patients had high PASI scores at week 0, with a mean of 34.9 (range 23.4–45). BSA in all patients was greater than 75% before initiating anti-IL17 biologic therapy.

Most patients in our study (13 patients, 81.2%) were treated with secukinumab; conversely, 3 patients were treated with ixekizumab (18.8%).

**Fig 2:**
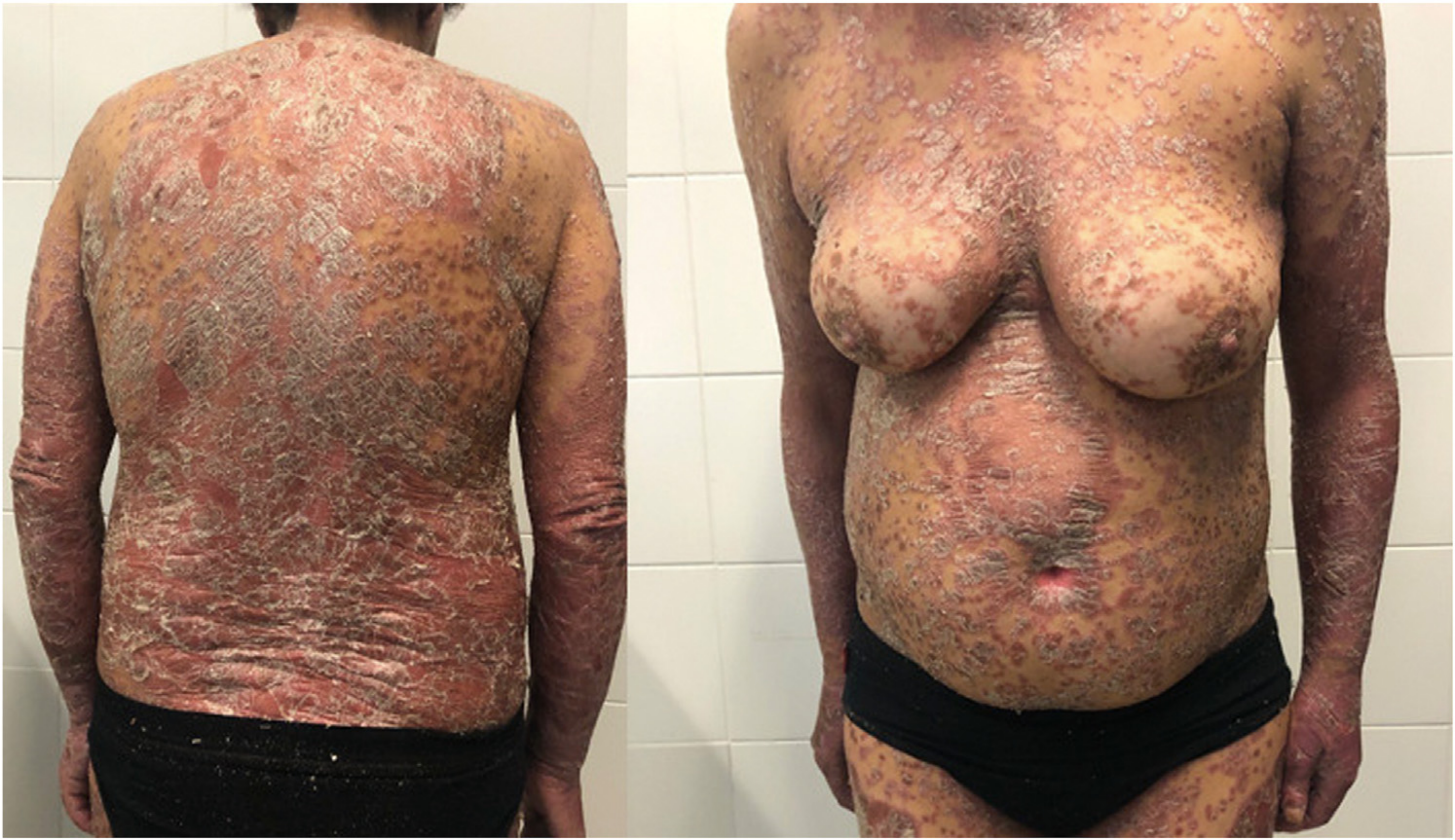
Erythrodermic psoriasis. Development of widespread, confluent erythema of the skin with scaling and pustules.

**Fig 3:**
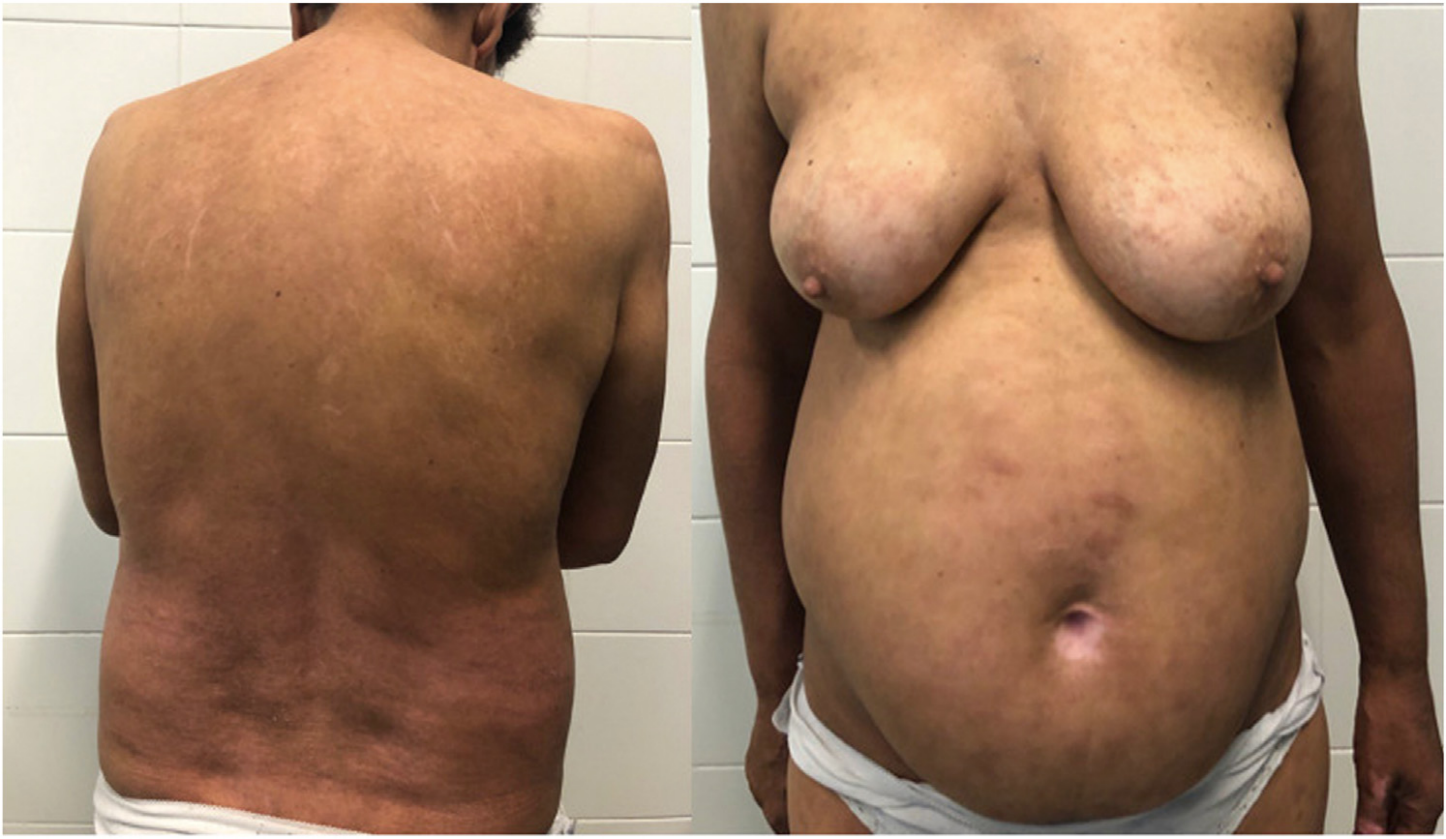
Patient response. Resolution of the erythroderma after 12-week therapy with Secukinumab.

Overall, the clinical response to both drugs was good (Figs 2 and 3). The primary endpoint of erythroderma clearance at week 12 was reached in 9 out of 16 patients (56.2%). Sub analysis for the single active principle, demonstrated a mean residual absolute PASI of 3.33 in patients treated with ixekizumab and 6.5 in those treated with secukinumab at week 12. The average time to clearance was 9 weeks for ixekizumab (range 4–16) and 14 weeks for secukinumab (range 4–24).

At week 24, other 6 patients had reached PASI 100, resulting in 93.7% overall stable response rate (15 out of 16 patients). Only one patient, treated with secukinumab had a residual PASI value of 14. However, the treatment was not discontinued as the initial PASI index was 45; thus, the response was considered satisfactory, and the patient eventually achieved complete clearance at week 36.

At week 36, 15 out of 16 patients were still in remission (93.7%), and a complete responder at week 12 had to discontinue due to psoriasis worsening and was switched to another drug.

At 52 weeks of therapy, 14 of 16 initial patients were on treatment with a complete response, as another patient receiving ixekizumab had a relapse and was switched to another drug.

At week 64, another patient relapsed and was switched to another drug; after 2 years, 13 of the 16 patients were still on therapy.

In summary, the rate of drug survival at 104 weeks was 81.2%. All patients demonstrated a consistent clinical response, with some very fast responders and only one showing slower improvement; otherwise, they remained cleared in the long term. However, 3 of 16 patients (18.7%) presented a relapse that led to anti-IL17 discontinuation, approximately weeks 36 and 64 in 2 patients on secukinumab therapy and week-52 weeks in one patient on ixekizumab therapy. All discontinuations were attributed to loss of drug efficacy. No adverse events or side effects were observed and compliance with the therapy was found to be 100%.

None of the volunteers contracted the Severe Acute Respiratory Syndrome- CoronaVirus 2 (SARS-CoV-2) infection or were forced to discontinue biological therapy. Moreover, post anti-SARS -CoV-2 vaccines administration, no patient showed disease recurrence or worsening.

## Discussion

The case series is representative of the natural history of this rare form of erythroderma, with onset in adulthood (mean age 52.5 years), more often in males (75%), with a sudden or gradual worsening of their psoriasis vulgaris (93.7%). These data are consistent with a Japanese study in EP patients treated with ixekizumab, whose mean age was 50.2 years with a male patient prevalence.^8^ Diverse factors have been associated with the worsening of psoriasis and erythroderma occurrence, including emotional stress, sunburn, infection, and medication, especially sudden discontinuation of steroids, cyclosporine, and methotrexate. A clear trigger in our patients was identified only in a minority of cases (25%), although very typical: infections in 2 cases and drug discontinuation in other 2.

According to the consensus of the National Psoriasis Foundation, EP treatment should consider the severity of the clinical situation, patient comorbidities, and accessibility to the drug of choice. First-line treatments are conventional drugs, such as cyclosporine, methotrexate, and acitretin, which are contraindicated in our patients or have already been administered without response. To address such unmet needs, recent evidence has emerged regarding the rapidity of action of new biological agents, particularly the anti-IL17 class. Interestingly, Th17 is the second most predominant T-cell type after Th2 in EP lesions.^10^ Subcutaneous administration is another advantage of parenteral infliximab, which is the only biological drug considered in these guidelines.

Present case series further supports high clinical improvement of both secukinumab and ixekizumab, with 56% PASI 100 at week 12 and 93.7% at week 24 in treatment of EP patients, offering additional information on long-term response and tolerability (52 and 104 weeks). A similar multicenter, retrospective study evaluated the use of secukinumab in 13 patients with EP; 53.9% achieved PASI 90 in 12 weeks. At week 52, 5 (38.5%) patients achieved PASI 90, 5 patients achieved PASI 100, and the median time to clearance was 3 weeks. No recurrence or adverse reactions were observed during the 52 weeks follow-up.^11^

Weng et al reported that 40% of patients treated with secukinumab were able to achieve PASI 90 at week 12, 70% patients responded to treatment, demonstrating evident clearing of psoriasis (PASI > 75) by week 16. At week 24, the percentage of patients achieving PASI 90 and PASI 75 decreased by 30 and 60%. One of these patients experienced relapse by week 24. Two (20%) patients demonstrated a sustained response after approximately 6 months.^6^

Mateu-Puchades and Mugheddu reported 100% of patients treated with secukinumab achieved PASI 90 at 16 (5 of 5 patients) and 8 weeks (3 of 3 patients), respectively.^7^^,^^12^

The safety and efficacy of ixekizumab in EP were evaluated in a Japanese study that reported the achievement of PASI75 by week 12 in all patients examined. Similar response rates were observed at week 24: 100.0% of patients maintained PASI75, 87.5% achieved PASI90 and 12.5% achieved PASI100.^13^ The achievement of a satisfactory long-term clinical response and a good safety profile in erythrodermic patients treated with ixekizumab was verified by other recent studies, demonstrating that the effects were sustained to week 244, the mean PASI score was 42.8 at baseline, 3.0 at week 52 and 5.0 at week 244.^8^^,^^14^^,^^15^

Rapid action is one of the most important requirements for treating these severe forms of psoriasis. Although our case series is limited, a direct comparison of the 2 anti-IL17 drugs depicts a faster onset of action for ixekizumab, with a mean of 9 weeks (range 4–16), compared with 14 weeks for secukinumab (range 4–24). This finding reflects similar results in psoriasis vulgaris, wherein a meta-analysis reported a greater short-term efficacy of ixekizumab than that of secukinumab.^16^ However, another real-life comparison between secukinumab and ixekizumab in EP treatment found that was better performing, with PASI 90 and PASI 100 response rates achieved at week 12 in 58 and 42% of the patients, respectively. At week 48, 82% of the patients achieved PASI 90 and 54% PASI 100. Conversely, in the ixekizumab group, the responses achieved at weeks 12, 24, and 48 were definitely lower.^17^

Another very crucial characteristic of EP is the high rate of relapse, with very unstable general conditions, situation that occurred in 3 out of 16 patients (18.7%) in our survey. The loss of efficacy occurred in an unpredictable manner, shortly after complete response in 1 patient, between weeks 36, 52, and 64. We decided to switch to different drugs; however, in a recent case series of patients with prior failure to receive secukinumab, ixekizumab still demonstrated a rapid response as early as week 4, which might be considered in further studies.^18^

No patients experienced adverse events or side effects for either drug, verifying that they were well tolerated, even in clinically unstable patients with severe general conditions. It is noteworthy that secukinumab has been successfully administered in EP patients who had renal failure requiring dialysis, achieved PASI 100 at 8 weeks, and were followed up for at least 1 year without adverse reaction.^19^^,^^20^ However, side effects have been reported in the literature, with the most common being mild in severity, including hepatic dysfunction, infection, allergic reactions, and injection site reaction.^13^

None of the patients enrolled in our study contracted SARS-CoV-2 infection. They were particularly monitored for the initial warning that SARS-CoV-2 infection could lead to the worsening of psoriasis.^21^

Moreover, with the initiation of vaccination programs against SARS-CoV-2 infection, the enrolled subjects immediately received the first dose of the vaccine, as they were recognized as frail patients. Treatment was not suspended as vaccine administration decreased during the scheduled interval between drug injections. Eventually, they underwent strict follow-up as it is known that vaccines can trigger psoriasis worsening.^22^ None of our patients demonstrated worsening psoriasis following vaccination.

## Conclusions

In conclusion, EP is a rare, life-threatening variant of psoriasis that requires prompt intervention, although the recommended first-line treatment often presents with side effects and contraindications in patients with unstable comorbidities. This case series suggests that anti-IL17 biologic drugs are well-tolerated and effective options. Further studies are required to validate our results and refine the treatment guidelines.

## Conflicts of interest

None disclosed.
